# The prognostic value of pretreatment CA-125 levels and CA-125 normalization in ovarian clear cell carcinoma: a two-academic-institute study

**DOI:** 10.18632/oncotarget.7216

**Published:** 2016-02-06

**Authors:** Huimin Bai, Guisha Sha, Meizhu Xiao, Huiqiao Gao, Dongyan Cao, Jiaxin Yang, Jie Chen, Yue Wang, Zhenyu Zhang, Keng Shen

**Affiliations:** ^1^ Department of Obstetrics and Gynecology, Beijing Chao-Yang Hospital, Capital Medical University, Beijing, China; ^2^ Department of Obstetrics and Gynecology, Peking Union Medical College Hospital, Chinese Academy of Medical Sciences & Peking Union Medical College, Beijing, China; ^3^ Department of Pathology, Peking Union Medical College Hospital, Chinese Academy of Medical Sciences & Peking Union Medical College, Beijing, China; ^4^ Department of Pathology, Beijing Chao-Yang Hospital, Capital Medical University, Beijing, China

**Keywords:** clear cell carcinoma, ovary, CA-125, pretreatment, normalization

## Abstract

**Objectives:**

The present study investigated the clinical implications of pretreatment carbohydrate antigen 125 (CA-125) levels and CA-125 normalization in patients with ovarian clear cell carcinoma (CCC), and it provides useful information for the improvement of monitoring strategies for this lethal disease.

**Methods:**

The medical records of patients with ovarian CCC who had undergone primary staging surgery or cytoreductive surgery followed by systemic chemotherapy were retrospectively reviewed. A range of clinico-pathological parameters were collected and examined.

**Results:**

A total of 375 women were included in the analysis. FIGO stage (p < 0.001) was identified as the only significant prognostic factor for relapse. Residual tumor and advanced stage (p = 0.001 and p < 0.001, respectively) were identified as independent adverse factors for survival. The potential risk factors associated with elevated pretreatment CA-125 levels included advanced-stage disease, positive residual tumors and negative endometriosis (p < 0.001, p = 0.001 and p <0.001, respectively). Pretreatment CA-125 levels were not associated with relapse-free survival (RFS) or overall survival (OS) (p = 0.060 and p = 0.176, respectively). CA-125 normalization after chemotherapy exhibited a positive linear correlation with advanced stage (r = 0.97, p = 0.001) and residual tumor (r = 0.81, p = 0.027) and a negative relationship with 5-year RFS (r = −0.97, p = 0.002) and 5-year OS (r = −0.97, p= 0.001). Patients with CA-125 levels that normalized before cycle 2 of chemotherapy had a similar prognosis as patients whose CA-125 levels normalized prior to chemotherapy (RFS: p = 0.327; OS: p = 0.654). By contrast, patients with CA-125 levels that normalized after cycle 2 of chemotherapy or never normalized were significantly more likely to experience disease progression.

**Conclusions:**

Pretreatment CA-125 levels are not very useful for predicting clinical outcome. CA-125 levels following treatment are a valid indicator for treatment monitoring. CA-125 normalization after the completion of cycle 1 of chemotherapy represents a distinct inflection point for decreased RFS and OS.

## INTRODUCTION

The World Health Organization has defined clear cell carcinoma (CCC) of the ovary as a distinct histological type since 1973 [1]. This tumor type accounts for 15% of all epithelial ovarian cancer (EOC) cases [2], and the incidence might be even higher among Asian women [3-5]. CCC is one of the most aggressive and malignant types of tumors, and it has a poorer clinical outcome than other types of EOC due to its resistance to platinum-based chemotherapy, particularly in patients with advanced or recurrent disease [2, 6-8]. The Gynecologic Cancer Intergroup (GCIG) initiated a meta-analysis in 2010 and demonstrated that the median overall survival (OS) for stage III/IV CCC was only 21.3 months [9]. The 5-year OS rate for patients with recurrent CCC was as low as 22.5%, and the mean OS was only 25.3 months [10], consistent with our previous studies [11]. CCC has a unique developmental pathway and possibly originates from endometriosis [4]. However, a management and efficacy monitoring strategy specifically for ovarian CCC has not been established, in part due to the relative rarity of this tumor type. Therefore, ovarian CCC is treated and monitored in the same way as other histological subtypes of EOC.

Carbohydrate antigen 125 (CA-125) is a glycoprotein antigen that is a predominantly used in the clinical setting to evaluate tumor response and to predict relapse in patients with EOC [12-16]. However, the prognostic value of CA-125 in patients with ovarian CCC has seldom been addressed in the literature [17-19], and the conclusions have been controversial. In the present study, we investigated the clinical implications of pre- and post-treatment serum CA-125 levels in patients with ovarian CCC to provide useful information for the improvement of monitoring strategies for this lethal disease.

## RESULTS

### Clinico-pathological characteristics

A total of 375 women met the eligibility criteria during the study period and were included in this analysis, and 164 of these patients who developed recurrent or persistent disease after primary treatment were described previously [11]. Table 1 shows the demographic and clinico-pathological characteristics of the 375 women. The mean age at initial diagnosis was 50.8 years, and 191 of the cases (50.9%) were premenopausal. Ascites and EAOC were present in 139 (37.1%) and 132 (35.2%) patients, respectively. The FIGO staging was distributed as follows: 180 cases (45%) were stage I (Ia: 63; Ib: 4; Ic: 113), 43 cases (11.5%) were stage II (IIa: 9; IIb: 13; IIc: 21), 132 cases (35.2%) were stage III, and 20 cases (5.3%) were stage IV. Initial pelvic and para-aortic lymphadenectomy was performed in 259 patients (69.1%). Node involvement was present in 74 patients, including one woman who had a positive left supraclavicular lymph node and was exempted from lymphadenectomy. Thirty-eight patients (10.1%) had macroscopic residual disease (residual tumor > 1 cm) within the abdominopelvic cavity after the initial surgery. Platinum/taxane chemotherapy was performed on 264 patients (70.4%) as the front-line therapy, and the remaining 111 patients received conventional platinum-based regimens.

**Table 1:** Clinicopathologic characteristics of the 375 patients with ovarian clear cell carcinoma (CCC)

Parameter		Number of patient	Percent (%)
Age(Mean;range**)**		50.8 ± 10.7; (29–85)	
≤45		128	34.1
>45		247	65.9
FIGO Stage at diagnosis			
I		180	45.0
II		43	11.5
III		132	35.2
IV		20	5.3
Endometriosis			
+		132	35.2
−		243	64.8
Ascites			
+		139	37.1
−		236	62.9
Initial CRS^a^			
Optimal		337	89.9
Suboptimal		38	10.1
Initial lymphadenectomy			
+		259	69.1
−		116	30.9
LNM^b^			
+		74	28.6
−		185	71.4
First line chemotherapy			
Taxane and platinum		264	70.4
Conventinal platinum-based regimen		111	29.6
Pretreatment CA-125 (U/mL) (mean;range)		1066.1 ± 1945.2; (1.9-17171.5)	
≤ 35		79	21.1
> 35		296	78.9
Disease status at completion of primary chemotherapy			
NED^c^		343	91.5
Persistent disease	Stable	25	8.5
	Progressive	7	
Relapse interval (month)(mean;range)		21.5 ± 24.3; (2-153)	
RFS^d^ time(month)(mean;range)		41.3 ±39.3; (1-201)	
OS^e^ time(month)(mean;range)		47.4±39.3; (1-201)	
Status at the last contact			
NED		225	60.0
AWD^f^		80	31.3
DOD^g^		70	18.7

Note:

a cytoreductive surgery

b Lymph node metastasis

c No evidence of disease

d relapse-free survival

e Overall survival

f Alive with disease

g Dead of disease.

### The relationship between pretreatment (prechemotherapy) CA-125 levels and clinico-pathological characteristics

Pretreatment CA-125 levels were elevated in the majority (78.9%) of patients with CCC. The median value was 1066.1 U/mL ± 1945.2, although this value ranged widely, from 1.9 to 17171.5 U/mL. Table 2 shows potential risk factors related to pretreatment CA-125 elevation. Patients with early-stage (stages I and II) disease and EAOC without residual tumors (≤ 1 cm) exhibited a significantly lower risk of elevated pretreatment CA-125 values (> 35 U/mL) compared to patients with advanced stage (stages III and IV), negative EAOC, and positive residual tumors (> 1 cm) (p < 0.001, p < 0.001 and p = 0.001, respectively).

**Table 2:** The association between pretreatment CA-125 levels and clinico-pathologic characteristics for patients with CCC

Parameter	Pretreatment CA-125		P value^a^
	Elevated	Normal	
Age(Mean;range)			
≤45	99	29	0.587
>45	197	50	
FIGO Stage at diagnosis			
I+II	158	65	<0.001
III+ IV	138	14	
Endometriosis			
+	65	67	<0.001
−	231	12	
Ascites			
+	103	36	0.078
−	193	43	
Initial surgery			
Optimal	259	78	0.001^c^
Suboptimal	37	1	
Initial lymphadenectomy			
+	207	52	0.483
−	89	27	
LNM			
+	61	13	0.477
−	146	40	

Note:

a Chi-square test

b Logistic regression analysis

CI: Confidence intervals

d Fisher's exact test.

The median pretreatment CA-125 levels of patients with early-stage CCC, negative residual tumors, and EAOC were significantly lower than patients with advanced stage disease, positive residual tumors, and negative EAOC (early vs. advanced stage: 881.3 U/mL vs. 1337.1 U/mL, p < 0.001; negative vs. positive residual tumor: 925.5 U/mL vs. 2312.7 U/mL, p < 0.001; positive vs. negative EAOC: 738.6 U/mL vs. 1243.9 U/mL, p = 0.035).

### Status at last contact

Thirty-two (19.5%) patients had persistent disease, including stable disease (25 cases) and progressive disease (7 cases), after the completion of primary treatment. The remaining 342 patients showed no evidence of disease upon imaging examination. A total of 132 (7.0%) patients relapsed during the follow-up period (47.4 ± 39.3 months). The relapse interval was 21.5± 24.3 months and ranged from 2 to 153 months. The sites of recurrent and persistent disease included the abdomen or pelvis (109 patients), liver (49), lymph nodes (21), vaginal stump (20), spleen (19), chest cavity (5), lung (5), and other sites (38). Salvage treatments for the 164 patients with recurrent and persistent disease consisted of repeated CRS, salvage chemotherapy, and radiotherapy, which were performed in 86 (53.7%), 164 (100%), and 21 (12.8%) cases, respectively, and 14 (8.5%) of these patients regained tumor control. Seventy (18.7%) women had died of the disease by last contact. Eighty (31.3%) patients were alive but still had tumors, and 16 patients had end-stage cancer. A total of 225 (60.0%) cases survived without any evidence of residual tumor at the time of the last visit. These data were described in detail previously [11].

Patient CA-125 levels normalized either before chemotherapy (76 cases), between cycles 1 and 2 (69 cases), between cycles 2 and 3 (63 cases), between cycles 3 and 4 (50 cases), between cycles 4 and 6 (48 cases), or after 6 cycles (44 cases). A lack of normalization by the completion of primary treatment was exhibited in 25 patients.

### The prognostic significance of pretreatment CA-125 levels and CA-125 normalization in ovarian CCC

**Figure 1:**
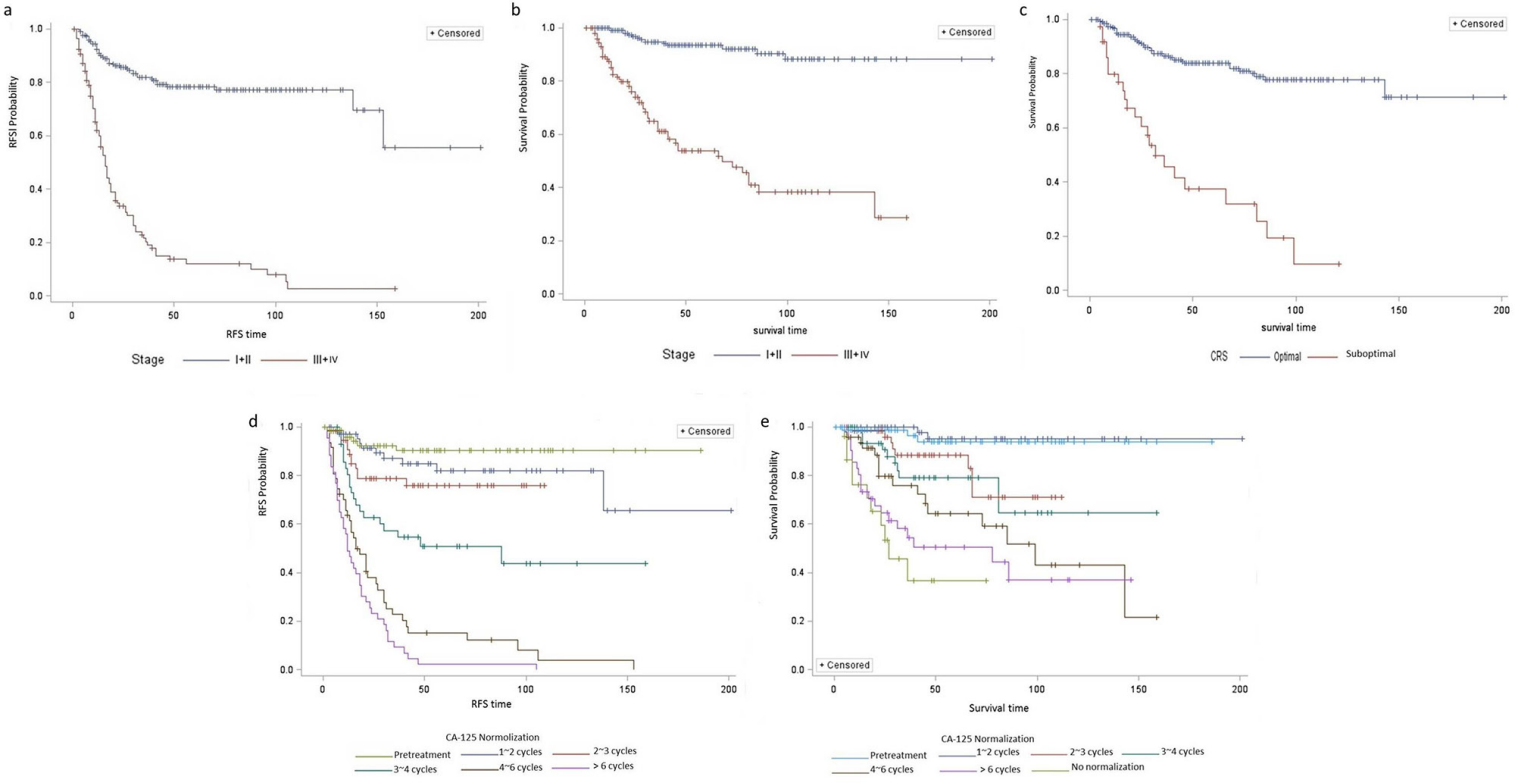
Risk factors related to relapse and survival Stage was the only significant prognostic factor for relapse (P < 0.001, panel a). Residual tumor and advanced stage (P = 0.001, and < 0.001, respectively; panel b and c) were identified as adverse factors for survival in the multivariate analysis. Serial CA-125 measurements during chemotherapy cycles were prognostic of disease progression and survival (panels d and e). The 5-year RFS rates were 90.1% and 82.1%, and the 5-year OS rates were 94.0% and 95.2% (RFS: p = 0.327; OS: p = 0.654), respectively, for patients whose CA-125 levels normalized prior to chemotherapy or between cycles 1 and 2. These two parameters successively decreased from 82.1% to 2.3% and from 95.2% to 50.1% in patients whose CA-125 levels normalized after cycles 2 ∼ 6 cycles, respectively. The 5-year OS rate was as low as 36.7% for patients who never attained normalized CA-125 levels.

The 5- and 10-year RFS rates were 56.0% and 49.7%, respectively, and the 5- and 10-year OS rates were 78.8% and 69.4%, respectively, for the entire series. Residual tumor and stage significantly impacted relapse rates in the univariate survival analysis (both p < 0.001; Table 3). Residual tumor, stage, and LNM were identified as risk factors for survival (P < 0.001, < 0.001 and = 0.039, respectively). Multivariate analysis revealed that stage was the only significant prognostic factor for relapse (P < 0.001). Residual tumor and advanced stage (P = 0.001, and < 0.001, respectively) were identified as adverse factors for survival in the multivariate analysis (Table 3 and Figure 1-a, b and 1c). The 5-year RFS rate in the advanced stage subgroup was as low as 12.1% vs. 78.5% in the early-stage subgroup. The 5-year OS rate for patients with advanced stage diseases and residual tumors was 37.7% compared with 94.5% for patients with early-stage diseases.

**Table 3:** Risk factors related to EFS and OS for patients with ovarion CCC

Parameter	Relapse^a^		P value^b^	P value^c^	HR^d^ (95% CI^e^)	DOD		P value^b^	P value^c^	HR^d^ (95% CI)
	+	−				+	−			
Age										
≤50	45	74	0.944			28	100	0.297		
>50	87	135				42	205			
Optimal CRS										
Optimal	111	207	<0.001			47	290	<0.001	0.001	2.48 (1.46-2.41)
Suboptimal	21	2				23	15			
FIGO Stage										
I+II	43	180	<0.001	<0.001	7.79 (5.36-11.33)	14	209	<0.001	<0.001	6.91 (3.72-12.84)
III+IV	89	29				56	96			
Endometriosis										
+	39	86	0.058			18	114	0.072		
−	93	123				52	191			
Ascites										
+	53	76	0.129			24	115	0.897		
−	79	133				46	190			
Lymphadenectomy										
+	92	144	0.827			46	213	0.407		
−	40	65				24	92			
LNM										
+	31	38	0.129			20	54	0.039		
−	61	107				26	160			
First line chemotherapy										
Taxane and platinum	96	144	0.557			50	214	0.835		
Other regimens	36	65				20	91			
Pretreatment CA-125 (U/mL)										
≤35	23	55	0.060			11	68	0.176		
>35	109	154				59	237			

Note:

a Thirty-two patients with persisitent disease were not included in this analysis

b Log rank test

c Cox proportional hazards model

d Hazard ratios

e Confidence intervals.

Pretreatment CA-125 levels and EAOC were not identified as significant predictors of RFS and OS. The clinical outcome of patients with elevated CA-125 levels was slightly inferior to that of patients with normal CA-125 levels (5-year RFS: 53.7% vs. 63.8%, p = 0.060; 5-year OS: 76.9% vs. 86.1, p = 0.176). By contrast, the prognosis for patients with positive EAOC was slightly better than for patients with negative EAOC (5-year RFS: 66.5% vs. 51.3%, p = 0.058; 5-year OS: 85.9% vs. 75.0, p = 0.072).

**Figure 2:**
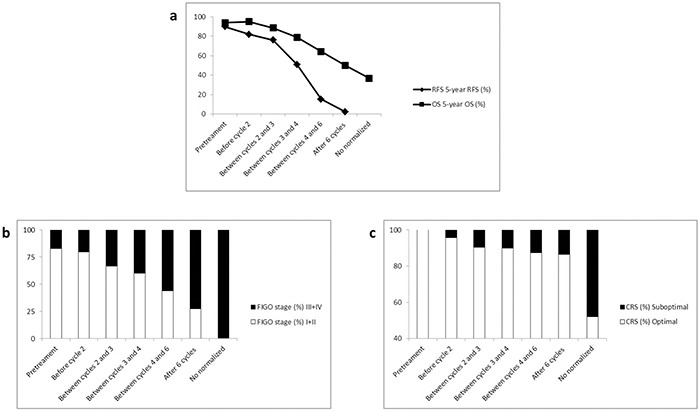
CA-125 normalization before the second cycle of chemotherapy is an inflection point when predicting relapse and survival in patients with CC Number of chemotherapy cycles for CA-125 normalization was positively correlated with advanced stage (r = 0.97, P = 0.001) and residual tumor (r = 0.81, P = 0.027), whereas it was negatively correlated with 5-year RFS (r = −0.97, P = 0.002) and 5-year OS (r = −0.97, P = 0.001; Figure 2). CA-125 normalization before chemotherapy cycle 2 was associated with a significantly higher rate of early-stage disease and negative residual tumor, which were identified as favorable factors for relapse and survival.

Pretreatment CA-125 levels were not associated with patient prognosis. However, serial CA-125 measurements during chemotherapy cycles were prognostic of disease progression and survival (Table 4, Figure 1-d and 1e, and Figure 2). Patients whose CA-125 levels normalized between cycles 1 and 2 had a similar prognosis (RFS: p = 0.327; OS: p = 0.654) as patients whose CA-125 normalized prior to chemotherapy. By contrast, patients whose CA-125 levels normalized between cycles 2 and 3, between cycles 3 and 4, between cycles 4 and 6, after 6 cycles, or never normalized were significantly more likely to experience disease progression. The 5-year RFS rates were 90.1% and 82.1%, and the 5-year OS rates were 94.0% and 95.2%, respectively, for patients whose CA-125 levels normalized prior to chemotherapy or between cycles 1 and 2. These two parameters successively decreased from 82.1% to 2.3% and from 95.2% to 50.1% in patients whose CA-125 levels normalized after cycles 2 ∼ 6 cycles, respectively. The 5-year OS rate was as low as 36.7% for patients who never attained normalized CA-125 levels.

**Table 4:** The prognostic significance of CA-125 normalization in ovarian CCC

CA-125 normalization	No. (%)	FIGO stage (%)			CRS (%)			RFS		OS	
		I+II	III+IV	P value^a^	Optimal	Suboptimal	P value^b^	5-year RFS(%)	P value^c^	5-year OS(%)	P value^c^
Pretreament	76 (20.3)	63 (82.9)	13 (17.1)	Referrence	76 (100)	0 (0)	Referrence	90.1	Referrence	94.0	Referrence
Between cycles 1 and 2	69 (18.4)	55 (79.7)	14 (20.3)	0.623	66 (95.7)	3 (4.3)	0.103	82.1	0.327	95.2	0.654
Between cycles 2 and 3	63 (16.8)	42 (66.7)	21 (33.3)	0.027	57 (90.5)	6 (9.5)	0.008	76.2	0.013	88.7	0.030
Between cycles 3 and 4	50 (13.3)	30 (60.0)	20 (40.0)	0.004	45 (90.0)	5 (10.0)	0.009	51.0	<0.001	79.0	0.003
Between cycles 4 and 6	48 (12.8)	21 (43.8)	27 (56.1)	<0.001	42 (87.5)	6 (12.5)	0.003	15.2	<0.001	64.4	<0.001
After 6 cycles	44 (11.7)	12 (27.3)	32 (72.7)	<0.001	38 (86.4)	6 (13.6)	0.002	2.3	<0.001	50.1	<0.001
No normalized	25 (6.7)	0 (0)	25 (100)	<0.001**^b^**	13 (52.0)	12 (48.0)	<0.001	-	-	36.7	<0.001

Note:

a Chi-square test

b Fisher's exact test

C Log rank test.

The rates of early-stage disease and negative residual tumor were markedly higher in patients whose CA-125 levels normalized before chemotherapy or between cycles 1 and 2 compared with patients who normalized after chemotherapy cycle 2 and failed to normalize during first-line chemotherapy, when the data were stratified by CA-125 normalization after chemotherapy. Linear correlation analysis also demonstrated that the number of chemotherapy cycles for CA-125 normalization was positively correlated with advanced stage (r = 0.97, P = 0.001) and residual tumor (r = 0.81, P = 0.027), whereas it was negatively correlated with 5-year RFS (r = −0.97, P = 0.002) and 5-year OS (r = −0.97, P = 0.001; Figure 2). CA-125 normalization before chemotherapy cycle 2 was associated with a significantly higher rate of early-stage disease and negative residual tumor, which were identified as favorable factors for relapse and survival. Therefore, CA-125 normalization before chemotherapy cycle 2 is a useful predictor for improved RFS and OS.

## DISCUSSION

### Clinico-pathological factors associated with elevated pretreatment CA-125 levels

Pretreatment CA-125 levels were elevated in the majority (78.9%) of patients with CCC in our series, which was consistent with the study by Tian and colleagues [17]. The potential risk factors related to elevated pretreatment CA-125 levels included advanced-stage disease, residual tumors after initial CRS and negative EAOC. For patients with these high-risk factors, the elevation rate and absolute value of pretreatment CA-125 levels were significantly higher compared with patients with early-stage disease, negative residual tumor and positive EAOC.

FIGO stage and optimal initial CRS are the two most important prognostic factors for patients with ovarian CCC [17, 24], similar to other cell types of EOC. Based on our data, the 5-year RFS rates and 5-year OS rates were 56.0% and 78.8%, respectively, for the entire series. The 5-year RFS rate for patients with advanced disease was as low as 12.1% compared with 78.5% for patients with early-stage disease when stratified by FIGO stage. The 5-year OS rate for patients with advanced stage diseases and positive residual tumors after initial CRS was only 37.7% compared with 94.5% for patients with early-stage disease. Kim et al. [19] also demonstrated that elevated CA-125 levels were a useful marker for predicting advanced stage disease, suboptimal debulking and platinum-resistance in patients with ovarian CCC, consistent with our analysis.

Ovarian CCC is one of the most common histological subtypes of EOC that is associated with endometriosis, with a frequency that ranges widely from 9% to 70% [22, 25-30], and endometriosis may increase the risk for this disease [26, 31-33]. EAOC presented in 35.2% of patients in our series. The present analysis confirmed our previous study [24] showing that EAOC negatively impacts the risk of elevated CA-125 and the absolute value of this biomarker in patients with CCC. The prognostic implications of endometriosis in ovarian CCC are controversial. Orezzoli [22] and Komiyama [28] found that patients with EAOC tended to have a favorable prognosis. Additionlly, the patient group in their study was likely younger with earlier stage disease [22]. Erzen et al. [25] demonstrated that EAOC exhibited lower stages at presentation and a better clinical outcome in patients with various histological subtypes of EOC, including CCC. This increased survival was evident in all age groups and all histological subtypes, and it could not be explained by better stage specific survival in any FIGO stage. Patients with EAOC in this analysis experienced slightly more favorable clinical outcomes compared with patients with negative EAOC (5-year RFS: 66.5% vs. 51.3%; 5-year OS: 85.9% vs. 75.0). However, this difference was not statistically significant (p = 0.058 and p = 0.072, respectively), consistent with our previous study [24]. Cutt et al. [26] also claimed that endometriosis *per se* is not predictive of prognosis in ovarian CCC. Therefore, the prognostic implication of endometriosis for ovarian CCC remains unclear, and further investigation will be necessary.

### The prognostic value of CA-125 normalization in ovarion CCC

The likelihood of elevated pretreatment CA-125 as well as pretreatment CA-125 levels themselves both increased significantly in patients with adverse prognostic factors, including advanced stage and positive residual tumor, whereas they decreased in patients with EAOC, a potentially favorable factor. However, absolute value of pretreatment CA-125 level was not identified as a predictor of relapse and survival based on our data or previous reports [17, 19]. Serial CA-125 measurements during chemotherapy treatment were indeed prognostic, and normalization before the second cycle of chemotherapy was associated with a significantly decreased risk of relapse and death. By contrast, patients with CA-125 levels that normalized after the completion of cycle 1 or never normalized were significantly more likely to experience disease progression. The 5-year RFS and OS rates in the current study were 90.1% and 94.0%, respectively, for patients whose CA-125 level normalized either before chemotherapy or before cycle 2. These two parameters successively decreased from 82.1% and 95.2% to 2.3% and 50.1% when CA-125 normalized before cycle 2 compared with after cycle 6, respectively. These two parameters successively decreased respectively from 82.1% and 95.2% to 2.3% and 50.1% in patients whose CA-125 levels normalized after cycles 2 ∼ 6 cycles, respectively. Furthermore, the 5-year OS rate was as low as 36.7% for patients who never normalized their CA-125 levels. No normalization of CA-125 after the completion of first-line chemotherapy was a sufficient condition for persistent disease. Therefore, this patient group was excluded from RFS-related analysis.

The prognostic value of CA-125 normalization before and after chemotherapy is well characterized in patients with EOC. Meyer et al. [34] concluded that CA-125 normalization at the third cycle of chemotherapy carried the greatest prognostic significance of any measurement during the treatment of primary disease. Zorn et al. [35] demonstrated that CA-125 normalization prior to chemotherapy was an independent predictor of PFS in patients with advanced EOC, particularly when tumors were debulked to a microscopic residual tumor and in the serous or endometrioid subtypes. Finally, CA-125 normalization before the second cycle of chemotherapy was associated with a decreased risk of death in an ancillary analysis of a phase III study of women with advanced serous ovarian carcinoma [16].

### CA-125 normalization before the second cycle of chemotherapy is an inflection point when predicting relapse and survival in patients with CCC

The prognostic value of CA-125 normalization in patients with ovarian CCC has not been well studied. Tian et al. [17] compared clinical outcome in patients with advanced CCC whose CA125 levels normalized to patients whose CA125 levels failed to normalize by the end of treatment. They concluded that CA-125 normalization by the end of treatment could be a valid indicator of RFS and OS, and likely reflected the degree of inherent chemosensitivity. However, the predictive role of CA-125 normalization was not stratified or compared with different cycles of chemotherapy in that study, likely due to their relatively small sample size (only 77 patients had CA-125 data recorded during treatment). Our analysis revealed that patient outcome grew worse as CA-125 normalization came later and later in the chemotherapy cycles. Indeed, CA-125 normalization before chemotherapy cycle 2 was a distinct inflection point for RFS and OS.

The rates of early-stage disease and negative residual tumor were markedly higher in patients whose CA-125 levels normalized before chemotherapy cycle 2 than in patients who normalized after the completion of chemotherapy cycle 1, when stratified by CA-125 normalization after chemotherapy. Linear correlation analysis also demonstrated that CA-125 normalization was positively correlated with advanced stage and suboptimal CRS, whereas it was negatively correlated with 5-year RFS and 5-year OS. CA-125 normalization before chemotherapy cycle 2 was associated with significantly higher rates of early-stage disease and negative residual tumor, which were identified as favorable factors for relapse and survival. Therefore, CA-125 normalization before chemotherapy cycle 2 may be a useful predictor for improved RFS and OS.

This analysis included results from 375 cases with ovarian CCC, making it one of the largest studies on this rare disease, despite its retrospective nature. Patient clinico-pathological data and follow-up information in the present study were relatively complete. These strengths enabled us to perform a robust analysis to evaluate the prognosis-predicting value of CA-125 normalization, and our findings provide useful information to improve monitoring strategies for this lethal tumor.

## MATERIALS AND METHODS

The medical records of all CCC patients diagnosed and treated at Peking Union Medical College Hospital (PUMCH) and Beijing Chao-Yang Hospital between 1993 and 2013 were collected and reviewed. Patients whose tumor specimens from the initial surgery were histologically confirmed as pure-type ovarian CCC were included for further analysis. Patients suffering from a primary malignant tumor in another part of the body or other malignant ovarian cell types were also excluded. Patient information, including demographic and pathological characteristics, surgery, subsequent systemic chemotherapy, and disease status at last contact, were collected and evaluated.

Serum CA-125 levels were measured using a radioimmunoassay kit (Roche F170 Modular system). Rising CA-125 levels were defined as a progressive increase in three consecutive serum antigen values above 35 U/mL, which is the commonly accepted normal upper limit for CA-125. Serum CA-125 served as both a pre- and post-operative tumor marker, and levels were evaluated within one week prior to staging surgery or cytoreductive surgery (CRS), prior to each cycle of chemotherapy, and at each contact during the follow-up period.

The surgical procedures, subsequent systemic chemotherapy, and the follow-up strategy after the completion of treatment were carried out as previously described [11]. Briefly, the predominant initial surgical procedure consisted of cytoreductive surgery or staging surgery. Ascites or washings were routinely collected before surgery, and cytological data were evaluated for all of the patients. Lymphadenectomy was not mandatory. Two independent pathologists with extensive experience in gynecological pathology reviewed all of the pathological slides for this analysis, and these pathologists were blinded to patient outcome. Disease staging was re-assigned using the exact FIGO staging criteria [20]. Optimal CRS was defined as abdominopelvic residual disease ≤1 cm after debulking surgery. Endometriosis-associated ovarian carcinoma (EAOC) was defined as the co-existence of CCC with endometriosis in the same and/or contralateral ovary and/or the co-existence of CCC with extraovarian endometriosis [21, 22].

Taxane/platinum or conventional cis/carboplatin-based chemotherapy (6 to 9 cycles) were administered as the post-operative first-line treatment. Responses to the systemic agents were recorded using version 1.1 of the Response Evaluation Criteria in Solid Tumors (RECIST) [23]. GCIG CA-125 response criteria [12] were used in the absence of measurable disease.

Relapse was documented using histological evidence of disease from tumor biopsies or fine-needle biopsies and/or the appearance of new lesions during imaging examinations. Relapse-free survival (RFS) times were calculated as the period between the date of initial surgery and the date of relapse. Women who were disease free at the time of their last visit were censored. OS times were calculated in months from the date of the initial surgery to the date of patient death from the disease. Patients who died from other conditions as well as patients surviving at the time of their last visit were censored.

Patient records and information were anonymized and de-identified prior to analysis; therefore consent was not necessary. The study protocol was approved by the ethics committee respectively at PUMCH and Beijing Chao-Yang Hospital, Beijing, China.

### Statistical analysis

All statistical analyses were performed using SAS® Version 9.2 (SAS Institute, Cary, NC). All of the tests were 2 sided, and P < 0.05 was considered statistically significant. Chi square or Fisher exact tests was performed to identify high-risk factors for elevated serum CA-125 levels. The two-tailed independent-sample Wilcoxon Rank Sum test was used to compare serum CA-125 levels between subgroups. The Kaplan-Meier method was used to analyze relapse and survival. A log rank test was used to compare the different survival curves. A Cox proportional hazards model was applied to all parameters that were significant in the univariate analysis. Simple lineal regression analysis was used to estimate linear correlations between CA-125 normalization and prognostic predictors.

## CONCLUSIONS

FIGO stage and residual tumor after initial CRS were the most important prognostic factors for ovarian CCC. CA-125 elevation rate and absolute value increased significantly in patients with advanced stage and residual tumor after initial CRS and decreased in patients with EAOC, which is itself a contested favorable factor for prognosis. However, CA-125 levels at pretreatment were not useful for predicting patient clinical outcome. CA-125 levels after treatment was a valid indicator for treatment monitoring. CA-125 normalization after the completion of cycle 1 of chemotherapy was a distinct inflection point for decreased RFS and OS.
